# Experimental Infection of Prairie Dogs with Monkeypox Virus

**DOI:** 10.3201/eid1104.040907

**Published:** 2005-04

**Authors:** Shu-Yuan Xiao, Elena Sbrana, Douglas M. Watts, Marina Siirin, Amelia P.A. Travassos da Rosa, Robert B. Tesh

**Affiliations:** * University of Texas Medical Branch, Galveston, Texas, USA

**Keywords:** monkeypox, rodent-borne, prairie dogs, orthopoxvirus, emerging viral disease, infectious disease, zoonoses, research

## Abstract

Studies of experimental infection of prairie dogs (*Cynomys ludovicianus*) with monkeypox virus are described. After intraperitoneal infection, all of the animals died within 11 days. Virus was cultured from their blood and oropharynx several days before death; at necropsy, most of the organs tested contained monkeypox virus. Marked hepatic and splenic necrosis were observed, along with mild inflammatory changes in the lungs. After intranasal (IN) infection, the primary pathologic changes were in the lungs and pleural cavity. Some of the IN infected animals (40%) survived, and monkeypox virus could be cultured from their nasal discharge and oropharynx for <22 days. Ulcerative lesions also developed on the lips, tongue, and buccal mucosa of the surviving animals. Our findings support an earlier report, which suggested that infected prairie dogs can transmit monkeypox virus by respiratory and mucocutaneous contact with susceptible animals and persons.

## Abstract 2

Infected prairie dogs can transmit monkeypox virus by respiratory and mucocutaneous contact with susceptible animals and humans.

In the summer of 2003, an outbreak of monkeypox virus infection occurred among humans living in the midwestern United States (*1**,**2*). A total of 32 human cases were confirmed. Most of the patients experienced a brief febrile illness with vesicular skin eruptions; no deaths resulted. Most patients with this illness had a history of contact with sick pet prairie dogs (*Cynomys* spp.), originally obtained from a single pet distributor (*1**,**2*). The epidemiologic investigation, clinical findings, and control of this outbreak were described (*1*).

A recent publication (*2*) reported the pathologic findings at necropsy in 2 sick prairie dogs confiscated during the 2003 outbreak. The animals showed necrotizing bronchopneumonia, conjunctivitis, and tongue ulceration. Virus isolation and electron microscopy examination of tissue samples from the animals demonstrated active viral replication in lungs and tongue, which suggested that both respiratory and mucocutaneous exposures are potentially important routes of monkeypox virus transmission from rodents to humans. To learn more about the nature of monkeypox virus infection in prairie dogs, we experimentally infected 10 of the animals with a human isolate from the 2003 U.S. outbreak.

## Materials and Methods

### Animals

Ten wild-caught, adult prairie dogs (*Cynomys ludovicianus*) were obtained from a commercial trapper, with permission of the Food and Drug Administration. Animals were housed in pairs in large (61 cm × 61 cm × 45 cm) metal cages within a Duo-Flow biosafety cabinet (Biochem Lab Products, Seaford, DE, USA) in an isolation room of an animal biosafety level 3 facility. All persons handling the animals had recently received a smallpox (vaccinia) vaccination and used appropriate personal protection. Animals were cared for in accordance with the guidelines of the Committee on Care and Use of Laboratory Animals (Institute of Laboratory Animal Resources, National Research Council) under an animal use protocol approved by the Institutional Animal Care and Use Committee at the University of Texas Medical Branch.

### Virus

The strain of monkeypox virus used (provided by the Centers for Disease Control and Prevention, Atlanta, Georgia, USA) was designated MPX 2003. This virus was originally isolated from a skin lesion of a person with monkeypox during the 2003 outbreak in the United States (*1*). A stock of the virus was prepared from infected Vero cells and was used to infect the rodents; the unsonicated frozen cell lysate had a titer of 10^6.1^ PFU/mL.

### Virus Assay

Tissues and other samples for virus assay were stored at –80°C. For analysis, the tissue samples were thawed and triturated in sterile TenBroeck glass tissue grinders in phosphate-buffered saline (PBS), pH 7.4, containing 30% heat-inactivated (56°C for 30 min) fetal bovine serum (FBS) to prepare a 10% tissue homogenate. After centrifugation at 5,000 rpm for 5 min to clarify the suspension, serial 10-fold dilutions from 10^–1^ to 10^–8^ were prepared in PBS containing 10% FBS. Similar dilutions were made with blood and throat swab suspensions for virus assay.

Dilutions of the tissue homogenates, blood, and throat swab suspensions were titrated in 24-well cultures of Vero cells, 4 wells per dilution, as described before (*3*). Cultures were incubated at 37°C, and plaques were counted 6 days later, as they were sharp and easy to read at that time. Virus titers were defined as the log of PFU per milliliter of sample.

### Experimental Infection of Animals

Since the response of prairie dogs to monkeypox virus infection was uncertain when these experiments were conducted, the animals were infected by the intraperitoneal (IP) or intranasal routes. Four rodents (MPX-1 to MPX-4) were injected IP with 10^5.1^ PFU of MPX 2003 virus. The other 6 animals (MPX-5 to MPX-10) were infected by the intranasal route; under Halothane (Halocarbon Laboratories, River Edge, NJ, USA) anesthesia, 2 drops (≈100 µL) of the stock virus solution containing 10^6.1^ PFU/mL were instilled into each nostril. After infection, all rodents were observed daily for signs of illness; if an animal died, a necropsy was performed, and tissues (liver, spleen, kidney, adrenal, pancreas, lung, heart, and brain) were taken for histopathologic examination and virus titration. Blood (100 µL from the retroorbital sinus) and an oropharyngeal swab were also taken daily from each animal for virus assay. The whole blood and the swab were expressed in 900 µL of PBS with 10% FBS. Twenty-five days after infection, the surviving animals were exsanguinated and euthanized, and a necropsy was performed to obtain tissue samples.

### Serologic Tests

Complement fixation (CF) tests were performed by microtechnique (*4*) with 2 full units of guinea pig complement and antigen titers >1:32. The antigen used in the CF tests was prepared from brains of infected baby mice injected intracerebrally with vaccinia virus; infected brains were treated by the sucrose acetone extraction method (*4*). CF antibody titers were recorded as the highest serum dilution giving +3 or +4 fixation of complement.

Plaque reduction neutralization (PRN) tests were conducted in 24-well microplate cultures of Vero cells, by using a technique described previously (*5*). The MPX 2003 virus was used at a dose of ≈20 PFU. Serial 10-fold dilutions of each serum specimen were incubated overnight at 5°C with the virus dose, before inoculation. Plaques were read on day 6 after inoculation; a 50% reduction of virus plaques, compared to uninfected control prairie dog serum, was used as the endpoint (*6*). PRN antibody titers were recorded as the highest serum dilution that produced ≥50% plaque reduction.

### Histopathologic and Immunohistochemical Methods

At necropsy, tissue samples were taken from the animals and preserved in 10% buffered formalin for 24 to 48 h, followed by storage in 70% ethanol. After fixation, the samples were processed for routine embedding in paraffin. Four- to 5-µm-thick tissue sections were made and stained by the hematoxylin and eosin (H&E) method (*5*).

Selected tissue sections were also studied immunohistochemically, by using vaccinia hyperimmune mouse ascitic fluid (1:100 dilution) as the primary antibody. A mouse-on-mouse IHC-ISO labeling kit (InnoGenex, San Ramon, CA, USA) was used, according to the manufacturer's instructions and a published protocol (*7*). The primary antibody was incubated with the sections at 4°C overnight. Tissue sections from an uninfected animal were used as negative controls.

## Results

### Clinical Manifestations

Two animals, MPX-2 and MPX-10, died of respiratory arrest during anesthesia and sample collection on days 5 and 6, respectively. They were omitted from the results.

Beginning on approximately day 4 after infection, most of the prairie dogs became lethargic and anorexic. The 3 animals that were infected IP (MPX-1, MPX-3, and MPX-4) died 8–11 days after infection. Visible lesions did not develop on the skin or mucous membrane of any of these animals.

**Figure 1:**
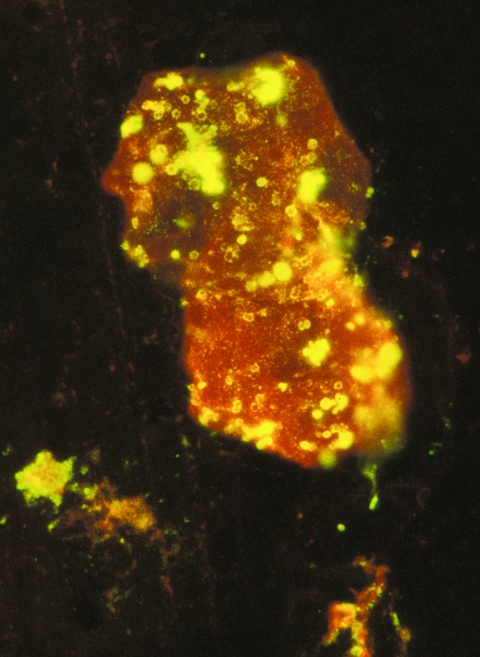
Photomicrograph of a smear of mucopurulent nasal discharge from a monkeypox virus–infected prairie dog (MPX-8), showing a swollen macrophage containing multiple fluorescein-labeled bodies (viral proteins) characteristic of poxvirus infection. Magnification ×1,400.

Three of 5 intranasally infected animals (MPX-6, MPX-7, and MPX-9) also died; their deaths occurred 11–14 days after infection. Their clinical symptoms (increasing lethargy and anorexia) were the same as those observed in the IP infected group. Two of the intranasally infected prairie dogs (MPX-5 and MPX-8) survived infection, although they were lethargic and anorexic for several days between weeks 1 and 2 after infection. In the latter 2 animals, vesicular lesions developed on their lips and tongue, along with nasal congestion and a mucopurulent nasal discharge. Inoculation of fresh samples of the nasal discharge into Vero cell cultures yielded monkeypox virus; and smears of the discharge, stained by immunofluorescence with a vaccinia hyperimmune mouse ascitic fluid, demonstrated swollen macrophages containing multiple fluorescent inclusion bodies, characteristic of poxvirus infection (Figure 1). The nasal congestion and discharge continued for ≈10 to 14 days, but it gradually diminished, and the 2 surviving prairie dogs appeared healthy when they were euthanized 25 days after infection.

### Virus Titrations

In the IP infected group (MPX-1, MPX-3, and MPX-4), monkeypox virus was first detected simultaneously in the blood and throat on day 5 or 6 after infection (Table 1). The amount of virus in these samples increased daily until death. In the IP infected group, the highest virus titers were found in liver and spleen; lower titers were observed in kidney and lung (Table 2).

**Table 1:** Results of virus titration of serial blood and throat swab samples taken from prairie dogs after experimental monkeypox infection

Day after infection	Animal no.*															
	MPX-1		MPX-3		MPX-4		MPX-5		MPX-6		MPX-7		MPX-8		MPX-9	
	B	TS	B	TS	B	TS	B	TS	B	TS	B	TS	B	TS	B	TS
1	0	NT	0	NT	0	NT	0	NT	0	NT	0	NT	0	NT	0	NT
2	0	0	0	0	0	0	0	0	0	0	0	0	0	0	0	0
3	0	0	0	0	0	0	0	0.7	0	0	0	0	0	1.0	0	0
4	0	0	0	0	0	0	0	0.7	0	0	0	2.4	0	0.7	0	1.6
5	0	0	0.7	0.7	1.9	1.9	0	2.2	0	0	0	2.6	0	1.5	0	1.7
6	1.2†	1.4	1.7	1.5	3.9	3.2	0	3.2	0	2.4	0	1.4	0	1.2	0	1.4
7	2.4	3.0	2.3	2.5	5.4	4.2	0	3.6	0	1.9	0	2.7	0	2.0	0	2.0
8	2.5	3.2	3.5	1.7	D	D	0	3.6	0	1.6	0	2.7	0	2.3	1.5	3.2
9	4.0	4.3	D	D			1.4	4.6	1.6	2.7	1.4	2.6	3.4	3.5	1.2	4.5
10	5.3	5.1					1.0	4.8	3.5	3.0	2.1	3.7	4.4	2.5	3.6	5.0
11	D	D					1.4	5.0	D	D	1.9	3.9	4.0	4.4	3.8	5.1
12							1.2	5.1			2.3	4.7	4.4	4.7	4.3	5.2
13							2.4	5.1			2.0	3.5	4.0	5.1	D	D
14							NT	NT			D	D	NT	NT		
15							4.5	4.4					4.0	5.0		
16							NT	4.3					NT	5.0		
17							NT	3.2					NT	5.2		
18							NT	2.1					NT	5.0		
19							NT	2.9					NT	4.8		
20							NT	1.7					NT	4.2		
21							NT	1.0					NT	4.0		
22							NT	0					NT	2.9		

*Animals MPX-1, MPX-3, and MPX-4 were infected intraperitoneally; animals MPX-5 to MPX-9 were infected intranasally. †Indicates titer of monkeypox virus in sample; log_10_ PFU/mL of sample (blood [B] or throat swab diluent [TS]); 0, <10^0.7^ PFU/mL; NT, not tested; D, animal dead.

**Table 2:** Monkeypox virus titers in selected tissues of experimentally infected prairie dogs at death

Route of infection	Animal no.	Virus titer (log_10_ PFU/g of tissue)*					
		Liver	Spleen	Kidney	Lung	Heart	Brain
Intraperitoneal	MPX-1	8.1	7.2	4.3	6.7	0	0
	MPX-3	7.6	7.4	5.4	6.8	3.5	3.6
	MPX-4	7.7	8.4	5.4	7.1	5.5	NT
Intranasal	MPX-5	0	0	0	0	0	NT
	MPX-6	0	0	0	0	0	4.7
	MPX-7	0	0	0	0	0	0
	MPX-8	0	0	0	0	0	NT
	MPX-9	3.0	2.4	2.4	8.0	4.5	0

*0, <0.7 (<10^0.7^); NT, not tested.

The temporal appearance, organ distribution, and amount of virus present in the blood, throat, and tissues of the intranasally infected prairie dogs were quite different. Among the 3 animals with fatal infections (MPX-6, MPX-7, and MPX-9) in this group, monkeypox virus appeared in the throat several days before it was detected in the blood (Table 1). At death, animal MPX-9 had 10^8.0^ PFU/g of virus in lung; smaller amounts of virus were detected in liver, spleen, kidney, and heart (Table 2). In contrast, no virus could be detected in these same organs in animals MPX-6 or MPX-7 at death.

Likewise, monkeypox virus was detected in the throats of the 2 surviving animals (MPX-5 and MPX-8) for 19 and 22 consecutive days, respectively (Table 1). No samples were taken on day 24; but throat swabs taken from MPX-5 on days 23 and 25 were negative; and samples from MPX-8 were negative on day 23 but positive (10^0.7^) on day 25 (data not shown). In these 2 animals, a comparable level of viremia developed; but the duration was uncertain, since no blood samples were taken after day 15. However, blood and organ cultures from the 2 survivors were negative when they were euthanized and underwent necropsy 25 days after infection (Table 2).

### Antibody Formation

All of the blood samples taken from the infected prairie dogs (Table 1) were examined by CF test for monkeypox virus antibodies, by using a vaccinia antigen. Only 2 samples gave a positive reaction. Blood (serum) samples from the 2 survivors, MPX-5 and MPX-8, both had a CF antibody titer of 1:64 on day 25 after infection, when the animals were euthanized. The last previous blood samples from these animals were taken on day 15 and were negative, so seroconversion occurred during week 2 or 3 after infection.

Serum specimens from MPX-5 and MPX-8, collected 25 days after infection, were also tested by PRN. Using 50% plaque inhibition as an endpoint, both animals had a neutralizing antibody titer of 1:320 against monkeypox virus.

### Histopathologic Changes

No histologic abnormalities were noted in the heart, pancreas, kidneys, or adrenal glands of any of the animals, regardless of the route of infection. The gastrointestinal tracts of a few animals were examined and were likewise unremarkable.

### Animals Infected Intraperitoneally

**Figure 2:**
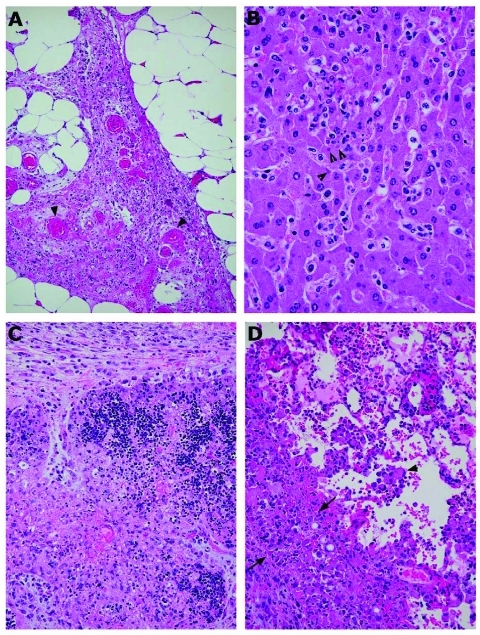
Photomicrographs of pathologic changes in tissues of prairie dogs infected with monkeypox virus. A) Abdominal adipose tissue from an intraperitoneally infected animal showing focal vasculitis (arrowheads), necrosis, and proliferation of fibroblasts. B) Mild hepatitis, characterized by focal inflammatory cell infiltration in the lobules and hepatocytes containing cytoplasmic inclusion bodies (arrowheads). C) Severe necrosis of the thymus from an animal infected intranasally. The necrotic areas contain many swollen macrophages with cytoplasm full of intensively eosinophilic material (viral proteins). D) Lung from the same animal, showing inflammation with swollen cells (arrowheads), alveolar edema, and necrosis of the pleura. The latter is infiltrated by many inclusion-filled macrophages and proliferating fibroblasts (the thick layer between the 2 arrows). Magnification: A, 10× objective; B, 40× objective; C, 10× objective; D, 20× objective. Hematoxylin and eosin stain.

At necropsy, firm nodular changes were observed in the abdominal adipose tissue (omentum) of the animals in this group (MPX-1, MPX-3, and MPX-4). Microscopically, multifocal necrosis of adipose tissue occurred, with vasculitis, prominent fibroblast proliferation, and infiltration by macrophages and other inflammatory cells (Figure 2A). Their spleens showed moderate to severe necrosis, mainly in the lymphoid areas as described before (*3*). The livers showed centrilobular necrosis, with some inflammatory cellular infiltration and prominent inclusion bodies in the hepatocytes (Figure 2B). The lungs exhibited mild-to-moderate thickening of the interstitium, with increased infiltration by mononuclear inflammatory cells.

### Animals Infected Intranasally

No evident hepatic lesions or splenic necrosis was observed in the 5 animals infected intranasally (MPX-5 to MPX-9). One of the animals (MPX-9) had marked inflammation and necrosis of adipose tissue and skeletal muscle, with proliferation of large fibroblasts and macrophages. The mediastinal lymph nodes, and thymus in some animals, exhibited marked lymphoid necrosis and depletion, with the infiltration by plump inflammatory cells containing dense eosinophilic material (Figure 2C). The lungs of animals MPX-6, MPX-7, and MPX-9 showed marked edema, hemorrhage, and necrosis, which also involved the pleura and muscle of the diaphragm. Extensive adhesions and a proliferation of swollen cells containing globules of eosinophilic material, later shown by immunohistochemical tests to be viral antigen, were evident in these structures (Figure 2D).

The 2 surviving animals in this group (MPX-5 and MPX-8) were euthanized on day 25 in apparent good health. Few histologic abnormalities were noted at necropsy, except for focal inflammation in the skin (lymphocytic infiltration in dermis). An immunostain of the skin for viral antigen was negative. Animal MPX-8 also had multifocal inflammation of the lung with epithelial and giant cell granulomas.

### Immunohistochemical Analysis

Selected tissue sections were studied immunohistochemically (IHC), by using a vaccinia mouse hyperimmune mouse and an IHC-ISO labeling kit. Tissue sections from 2 uninfected prairie dogs were used as negative controls. No positive staining was observed in the control animals. Likewise, tissues from the 2 survivors (MPX-5 and MPX-8) were also IHC-negative.

### Animals Infected Intraperitoneally

In the liver of IP infected animals (MPX-1, MPX-3, and MPX-4), most of the inclusion bodies were strongly IHC-positive for poxvirus antigen. Depending on the severity of the histologic abnormality, this positive staining sometimes involved the surrounding cytoplasm and cellular membranes. Some of the smaller inclusion bodies were negative. The spleen was also IHC-positive, corresponding to the severity of pathologic changes. In some animals, the cells lining the surface of the splenic capsule (mesothelial cells) were enlarged or tall and stained strongly positive for viral antigen. In these animals, the positive staining also appeared to involve the superficial zones of the neighboring organs such as the pancreas and adrenals, which were otherwise negative and without pathologic changes. This pattern suggested direct virus spread between adjacent sites when the boundaries (capsules) were broken. Necrotic areas in the perisplenic and periadrenal fat also stained strongly positive.

### Animals Infected Intranasally

**Figure 3:**
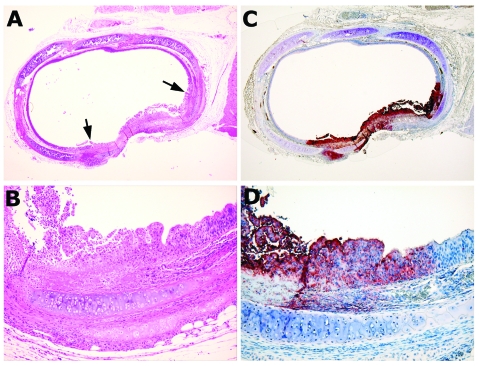
Bronchus from animal MPX-9, which was infected intranasally. A) Cross-section of a bronchus, showing focal metaplasia and proliferation (between the arrows) of the luminal epithelium. B) Higher magnification showing the details of the metaplastic epithelium, accompanied by focal necrosis. Compare to the adjacent unaffected area, which is lined by normal ciliated columnar epithelial cells. C and D) Immunohistochemical staining of the corresponding field shows presence of viral antigen limited to the region of epithelial abnormality. A and B, hematoxylin and eosin stain; C and D, immunoperoxidase staining with vaccinia antibody. Original magnification: A and C, 4× objectives; B and D, 20× objectives.

In animal MPX-9, viral antigen was present in the liver, lungs, mediastinum, and bronchus. The mediastinal lymph nodes were also strongly positive, accompanied by central necrosis. An antigen-positive area in a bronchus of this animal showed distinctive epithelial cell proliferation and squamous metaplasia, as opposed to a negative zone, which was lined by normal columnar epithelium (Figure 3).

Although no evidence of hepatocytic degeneration or necrosis was seen in the H&E-stained sections of liver from the other 4 animals in this group (MPX-5 to MPX-8), the immunostain for viral antigen highlighted rare inclusion bodies in the hepatocytes of animals MPX-6 and MPX-7, which indicated a small amount of monkeypox virus replication in the liver. The spleens of these 4 animals were negative for monkeypox virus antigen by IHC. Scattered antigen-positive interstitial cells (mostly macrophages) were observed in lungs of animal MPX-6; but lungs in the other animals (MPX-5, MPX-7, and MPX-8) were negative by IHC. Sections of the adrenals, kidneys, and heart of all of the infected animals were likewise negative.

## Discussion

Results of this study confirm that prairie dogs are highly susceptible to infection with monkeypox virus, although the observed death rate and pathologic changes were less severe than in 13-lined ground squirrels (*Spermophilus tridecemlineatus*) that had been infected IP and intranasally with the same virus dose (*3*). In contrast to ground squirrels, the pathogenesis and severity of monkeypox virus infection in prairie dogs varied, depending on the route of infection. The IP infected prairie dogs all died after infection; at necropsy, marked hepatic and splenic necrosis was seen, along with mild-to-moderate inflammatory changes in the lungs. Only 3 of the 5 intranasally infected prairie dogs died. In 1 of these animals (MPX-9), the virologic, histopathologic, and immunohistochemical findings were similar to those observed in the IP infected prairie dogs. However, in the other 2 animals with fatal infections (MPX-6 and MPX-7), few pathologic changes were observed in the liver, spleen, or other abdominal organs, although marked edema, hemorrhage, and necrosis were observed in the lungs. The reason for this different response is unknown, but our sample size was small.

The pattern of monkeypox virus infection seen in the 2 surviving prairie dogs (MPX-5 and MPX-8) was potentially the most important. These 2 animals continued to have infectious monkeypox virus in their throat and nasal discharge for several weeks after infection.

The pattern of experimental infection in the intranasal group of prairie dogs concurs with the clinical and pathologic observations made during the 2003 monkeypox virus outbreak in the United States. Guarner et al. (*2*) reported that 10 (67%) of 15 prairie dogs in 1 affected pet store died rapidly; the other 5 animals exhibited anorexia, wasting, sneezing, coughing, swollen eyelids, and nasal discharge. Our intranasally infected animals manifested similar symptoms and had a comparable death rate (60%). Two of the sick prairie dogs at the affected pet store were euthanized, and necropsies were performed (*2*).

Apart from ulcerative lesions on the tongue and eyelids, these animals had bronchoalveolar pneumonia with edema, necrosis, and a marked infiltrate of macrophages containing many poxvirus particles. Mild inflammatory changes occurred in the liver and spleen, and other organs appeared normal. Although direct culture of monkeypox virus from tissues of these 2 animals was not attempted, the authors noted difficulty detecting monkeypox virus DNA by standard polymerase chain reaction (PCR) and by PCR followed by restriction-endonuclease fragment length polymorphism, even though viral antigen was easily detected by IHC in the same tissue samples (*2*).

Our experience was similar with animals MPX-6 and MPX-7. Despite the presence of viral antigen in the liver of these 2 experimentally infected prairie dogs, cultures of their lungs, livers, and spleens did not yield infectious virus. Yet on the day before death, monkeypox virus was isolated from their blood and throat swabs. Thus the exact cause of death in these 2 animals is uncertain.

In animals MPX-5 and MPX-8, cutaneous ulcerative lesions developed on the lips, tongue, and buccal mucosa, along with nasal congestion and discharge. The same clinical manifestations were reported from the affected pet prairie dogs (*2*). In view of the prolonged shedding of monkeypox virus in the throat and nasal discharge of animals MPX-5 and MPX-8, such pets likely could easily transmit virus by direct contact or by bite to cagemates or to humans handling them. Guarner et al. (*2*) concluded that both respiratory and direct mucocutaneous exposures are potentially important routes of transmission of monkeypox virus between rodents and humans. Our findings with experimentally infected prairie dogs support those conclusions.
